# Variation in activity rates may explain sex-specific dorsal color patterns in *Habronattus* jumping spiders

**DOI:** 10.1371/journal.pone.0223015

**Published:** 2019-10-16

**Authors:** Lisa A. Taylor, Collette Cook, Kevin J. McGraw

**Affiliations:** 1 Entomology and Nematology Department, University of Florida, Gainesville, Florida, United States of America; 2 Florida Museum of Natural History, University of Florida, Gainesville, Florida, United States of America; 3 School of Life Sciences, Arizona State University, Tempe, Arizona, United States of America; National Institute of Biology, SLOVENIA

## Abstract

In many animals, color pattern and behavior interact to deceive predators. For mimics, such deception can range from precise (near-perfect mimicry) to only subtle resemblance (imperfect mimicry) and such strategies often differ by sex because of differing ecological selection pressures. In this field study, we examine variation in behavior and ecology that may be linked with sex differences in dorsal color pattern in three sympatric species of *Habronattus* jumping spiders (*H*. *clypeatus*, *H*. *hallani*, *H*. *pyrrithrix*). Males of these species have conspicuous dorsal patterning that is subtly reminiscent of the general color patterns of wasps and bees, while females are cryptic. We show that, compared with females, these conspicuous males exhibited increased leg-waving behavior outside of the context of courtship; such behavior is common in jumping spiders that mimic wasps and bees presumably because a mimic’s waving legs resemble antennae. Males of a fourth sympatric species (*H*. *hirsutus*) without conspicuous dorsal patterning did not exhibit increased leg-waving. These results are consistent with and offer preliminary support for the idea that male color and behavior may work together to deceive predators. We also examined whether higher movement rates of males (who must wander to find females) and/or different use of the microhabitat by the sexes could explain sexual dichromatism. We found that microhabitat use was similar for males and females, but males of all three conspicuously-colored species spent more time actively moving than females. To our knowledge, this is the first study to speculate that conspicuous male dorsal coloration in *Habronattus* may have a deceptive function, and to explore why dorsal coloration differs between the sexes.

## Introduction

In many animals, morphology and behavior interact to facilitate evasion or deception of potential predators; as such, selection by predators is thought to act on particular combinations of these traits (e.g., butterflies [1], snakes [2], grasshoppers [3], hoverflies [4], moths [5]). For example, a variety of invertebrates are known to mimic ants; such mimics often use combinations of ant-like morphology and behavior, including erratic running and waving of their front legs in a way that mimics the waving of an ant’s antennae (reviewed in [6,7–10]). Also, hoverflies appear to mimic the general color patterns of wasps and bees, and in some cases even mimic wasp-like flight patterns [4]. Such examples range from precise, near-perfect Batesian mimicry (e.g., *Myrmarachne* jumping spiders that closely mimic ants [9,11]) to general or imperfect resemblance in color and behavior (e.g., *Syrphus* hoverflies that only subtly resemble wasps [4,12]).

Interestingly, such defensive strategies often evolve differently between the sexes. Although many of the best-studied examples of sexual dimorphism are traits shaped by sexual selection via mate choice or competition [13], sexual dimorphism can also be shaped by ecological selection, where males and females experience different selection pressures as a result of sex-specific differences in size, diet, habitat use, activity levels, thermoregulatory requirements, or predator exposure (reviewed in [14]). There are several examples of deceptive coloration (e.g., mimicry) evolving differently between the sexes (reviewed in [15]). For example, in butterfly mimicry complexes, females are typically the mimics; it has been suggested that slower-flying females need added protection from predators or that mimicry in males is less likely to evolve because a male’s species-specific color patterns are crucial for mate recognition (reviewed in [15]).

Jumping spiders (family Salticidae) are an excellent group in which to examine questions about sex differences in predator threats and potential deception. Across the family of more than 6000 described species [16], there are numerous species in which dorsal coloration differs dramatically between the sexes (e.g., [17]). Although visual signals are important in courtship displays in many jumping spider species (e.g., [18,19–21]), there are many cases in which the sexually dichromatic body regions are not overtly displayed and are actually oriented away from potential mates during courtship (pers. obs). In some of the most striking examples, one or both sexes of jumping spider appear to mimic brilliant and colorful hymenopterans, most notably wasps and bees including the so-called ‘velvet ants’ in the family Mutillidae ([22]; see S1a and S1b Fig). Color patterns are usually paired with behavioral mimicry, in which the spiders frequently wave their first pair of legs, presumably to resemble the antennal movement characteristic of hymenopterans (pers. obs., see also [7,8,9]).

**Fig 1:**
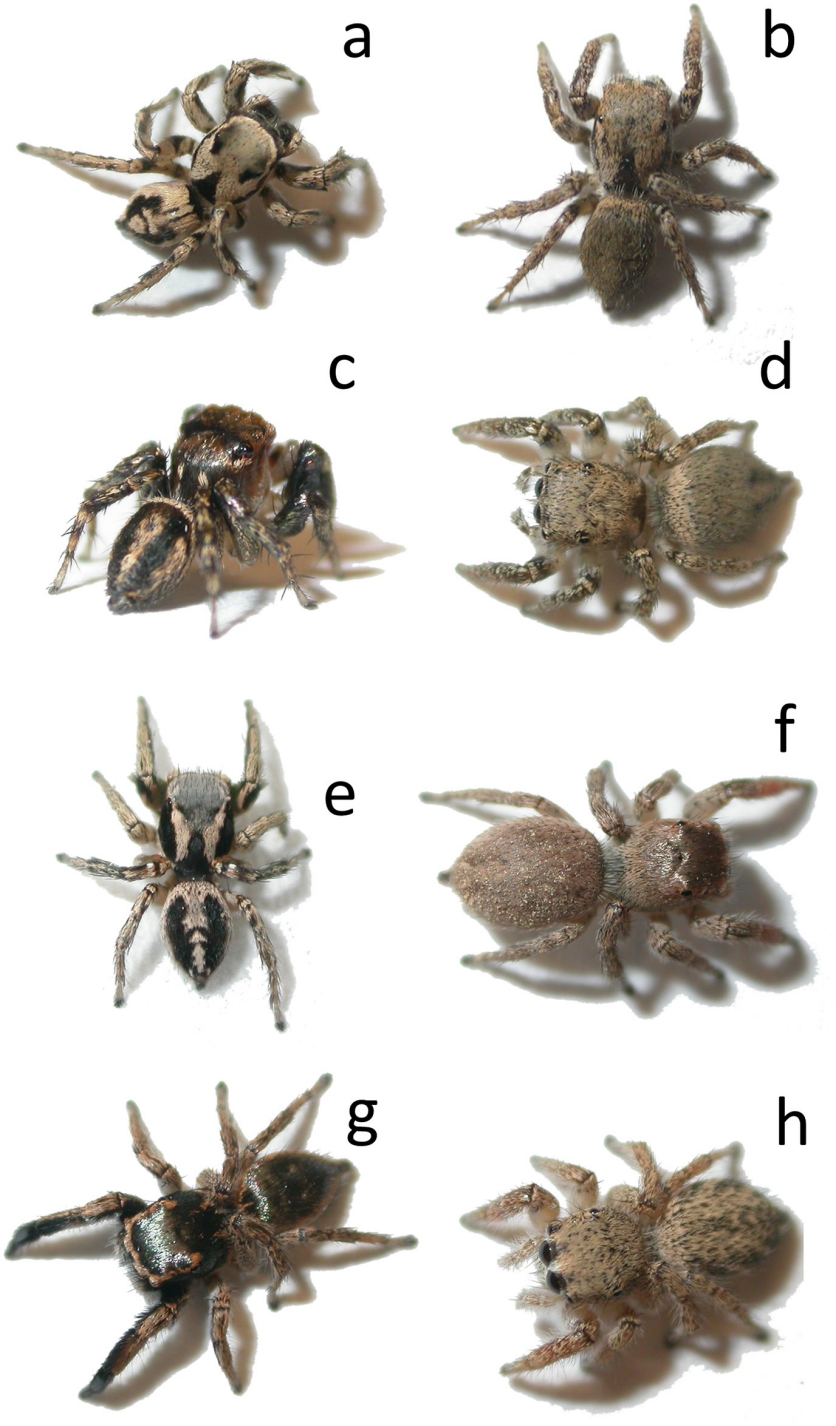
Sex differences in dorsal color pattern in four sympatric species of *Habronattus*. *H*. *clypeatus* male (a) and female (b), *H*. *hallani* male (c) and female (d), *H*. *pyrrithrix* male (e) and female (f), *H*. *hirsutus* male (g) and female (h). Note that males of *H*. *clypeatus* (a), *H*. *hallani* (c), and *H*. *pyrrithrix* (e) exhibit conspicuous dorsal patterning, while males of *H*. *hirsutus* (g) are solid dark gray/black. While females all look similar to one another, they can be identified based on subtle differences in dorsal and facial markings.

**Fig 2:**
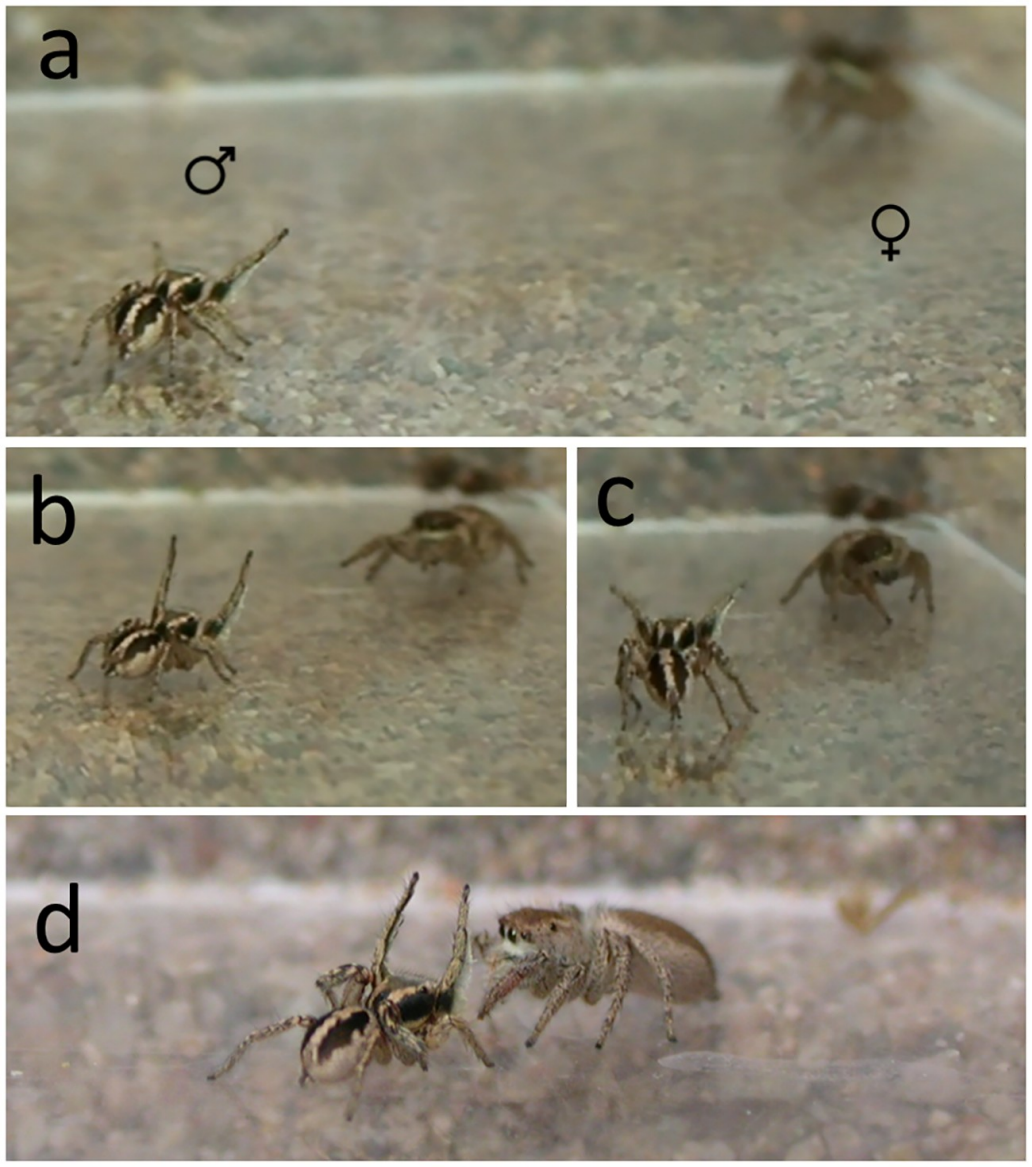
Courtship display in *Habronattus pyrrithrix*. In the first stage of display, the male approaches the female and displays his ornamented red face and the green undersides of his front legs (a, b, c). In stage 2 of display, he approaches and stops directly in front of the female and initiates an additional seismic component to his display (d). Note that the male’s conspicuous dorsal pattern is oriented away from the female throughout the entire display.

In this study, we investigated behavioral and ecological predictors of sexually dimorphic dorsal coloration in a community of three species of *Habronattus* jumping spiders. Most behavioral work on *Habronattus* has focused on the complex ornamentation on their faces and legs, their exuberant courtship displays, and the role of sexual selection in driving diversification of these traits (e.g., [20,23,24–26]). However, in many *Habronattus* species, males also have conspicuous dorsal color patterns consisting of bold black and white stripes or chevrons (see [27]; Fig 1a, 1c and 1e) that are visible from above and behind the spider, but which are not overtly displayed to females during courtship; in fact, these patterns are actually oriented away from the female during the entire courtship display (Fig 2). In contrast, females of most species lack these conspicuous markings and are instead drab and cryptically colored ([27], Fig 1). The striking male dorsal patterns subtly resemble the bold stripes on the dorsal surfaces of a wide range of common hymenopterans that are frequently seen in the leaf litter in the same habitat as these spiders and can inflict a painful sting (e.g., velvet ants: Family Mutillidae; ground-nesting bees like *Lasioglossum* spp.; pers. obs). While these male spiders don’t clearly resemble one particular model hymenopteran, their color patterns are reminiscent of hymenopterans in general. Leg-waving is a common feature of jumping spider communication and courtship [28], and this waving and extension of the first pair of legs occurs throughout the entire courtship display in *Habronattus* (Fig 2). Anecdotal observations of male behavior in the field suggested that these conspicuously patterned male *Habronattus* also frequently wave their front legs when moving through their habitat (outside of the context of courtship, and do so more than females) in a way that appears to enhance their resemblance to hymenopterans (pers. obs; S1 File (video)). Such leg-waving behavior (or false antennation) that occurs outside of the context of courtship is very common and pronounced among other salticids that clearly mimic hymenopterans (reviewed in [7,8,9]).

The preliminary observations described above led us to the hypothesis that these conspicuous male color patterns, combined with characteristic leg-waving behavior, are functioning as deceptive signals, either to directly (but imperfectly) mimic hymenopterans [29] or to exploit the perceptions of predators that have evolved to avoid such color patterns and behaviors [30]. Because these two hypotheses are difficult to disentangle (see discussion in [30,31]), we lump them together here as a single hypothesis, which we refer to as the ‘deception hypothesis’.

The present study has two main goals. First, we test initial predictions of the deception hypothesis described above, which will allow us to qualitatively weigh support against other alternative explanations for conspicuous male dorsal coloration in *Habronattus* (e.g., that it functions as disruptive coloration [32,33,34] or motion dazzle coloration [35,36,37], see additional details in the Discussion). We focus our study on three sympatric *Habronattus* species found within a single field site that all have males with conspicuous dorsal patterning (see ‘Study species’ section below). Because we are studying a small community of species, this is not intended to be a phylogenetic comparative study, but rather a behavioral study that is replicated across three sympatric species that are common at our field site to increase the robustness and generality of our conclusions. The deception hypothesis posits that conspicuous male color and leg-waving behavior function together to deceive potential predators. As such, it predicts that these conspicuously colored males should also exhibit correspondingly higher leg-waving rates in a non-sexual context compared with drab and cryptic females. Interestingly, there is a fourth species of *Habronattus* that is also common in this habitat (*H*. *hirsutus*) in which males do not have conspicuous coloration (both males and females are solid in color, although males are darker than females [27], Fig 1). This species is the most distantly related of the group [38] and thus we use it as a comparison for the other three. Because male *H*. *hirsutus* lack conspicuous markings, we predict to find comparatively less non-sexual leg-waving among males compared with females. To provide a context for understanding the selection pressures driving such color patterns, we report all predation events observed on all four species of *Habronattus*.

The second goal of this study was to address the question of why such conspicuous colors in *H*. *clypeatus*, *H*. *hallani*, and *H*. *pyrrithrix* are male-specific. Here we test two potential hypotheses: the ‘activity hypothesis’ and the ‘microhabitat hypothesis’. In the ‘activity hypothesis’, we posit that males and females are exposed to different selection pressures due to differences in their movement rates. Previous work with insects and fish has shown that movement and color pattern interact synergistically, such that cryptic coloration is only beneficial to motionless prey; if prey become active, the benefits of crypsis go away [39]. This suggests that, for an animal that is active, coloration strategies other than crypsis may be more adaptive [40–42]. In a phylogenetically controlled study of butterflies, Merilaita and Tullberg [43] found that aposematic and mimetic coloration were more likely to evolve in butterflies that are active during the day, whereas nocturnal species that rest during the day were more likely to be cryptic. This suggests that high rates of activity may constrain the evolution of crypsis [42,43]. We know of two other studies that have examined sex differences in movement patterns in free-ranging jumping spiders; in both cases males spent more time moving and females spent more time feeding [44,45]. In a concurrent study of misdirected courtship in our focal population, we observed males wandering in search of mates and initiating courtship whenever they found them [46]. Such differences in behavior between the sexes may explain why females are cryptic, while males are not.

In the ‘different microhabitat hypothesis', we posit that males and females may face different selection pressures if they inhabit different microenvironments (e.g., the substrate and/or lighting conditions in which they spend most of their time). Sex-specific differences in habitat use have been argued to be a driving force in the evolution of sex differences in coloration in other animals (e.g., grasshoppers [47,48], isopods [49], killfish [50]). In the buprestid beetle *Chrysobothris humulis*, males, but not females, mimic distasteful chrysomelid beetles; Hespenheide [51] suggests that this may be a result of the fact that males spend their time in different habitats (i.e., legume twigs) where there are a greater number of their chrysomelid models and where they are exposed to higher predation compared with females, which exhibit metallic green coloration. It could be that male and female *Habronattus* differ in dorsal coloration due to differences in the background colors and light levels of their preferred microhabitat habitat types (e.g., leaf litter, vegetation, rocks, and dirt) or due to differences in the suites of potential predators or models in those different microhabitats.

Similarly, we posit that males and females may face different selection pressures because males traverse a wider variety of habitat during mate-searching while females can wait for suitors to approach them and can therefore remain in a more homogeneous habitat. Empirical work with birds and artificial prey has shown that, in heterogeneous habitats, survival is maximized if prey have color pattern that are intermediate in their match between the different microhabitats, rather than a close match to either one [42,52]. Using an evolutionary simulation, Merilaita and Tullberg [43] found that, if animals had to move through multiple habitats of different colors, then alternatives to crypsis (i.e., aposematic coloration) were more likely to evolve, suggesting that habitat heterogeneity may constrain the evolution of crypsis. In the jumping spider *Phidippus clarus*, females showed higher site fidelity than males [44]; thus it’s plausible that females experience less habitat heterogeneity. If *Habronattus* females simply have to wait for males to find them, they may be able to remain in a single microhabitat type where they are well-suited for cryptic coloration. In contrast, if males need to seek out females, they may have to travel greater distances through a larger number of habitat types, reducing the effectiveness of crypsis and making deceptive coloration more adaptive.

The ‘activity hypothesis’ and the ‘different microhabitat hypothesis’ are not mutually exclusive, yet each generates specific and testable predictions. Here we weigh support for each using direct behavioral observations of free-ranging individuals in the field. To our knowledge, only three studies have examined salticid movement patterns using focal observations on free-ranging spiders [44–46], and no study has attempted to address hypotheses to explain sex differences in salticid dorsal coloration that is not involved in courtship.

## Methods

### Study species

The genus *Habronattus* includes approximately 100 species, primarily in North America, with a diversity of elaborate visual ornaments and dramatic multimodal courtship displays [27,38]. In addition to the colorful ornaments that males display to females (most often on their faces and front legs), males and females of many species have strikingly different dorsal coloration [27]. Although some species of *Habronattus* are known to hybridize [53], the four species examined in this study are all from different species groups [38] and do not hybridize in the field or lab (pers. obs).

In *Habronattus clypeatus* (Banks), *H*. *hallani* (Richman), and *H*. *pyrrithrix* (Chamberlin), males have striking and contrasting patterns of black and white stripes and/or chevrons (Fig 1a–1f). In *H*. *hirsutus* (Peckham and Peckham), the dorsum of males is solid in color (Fig 1g and 1h). Geographic variation in coloration is common within the genus *Habronattus* (see [27]) and thus it should be noted that some subtleties of color pattern, as well as behavior, described here might be typical of this Phoenix, AZ population, and may vary across the species range. These four species are all relatively common in riparian areas and gardens around Phoenix.

### Study site

All observations of spider behavior were made at the Rio Salado Habitat Restoration Area in Phoenix, Arizona, USA (33.42°N, 112.07°W). The mission of this restoration area is to reestablish native wetland and riparian habitats that were historically associated with the Salt River (Rio Salado), which used to flow year-round [54]; thus it is an ideal natural habitat to study the natural history and behavior of *Habronattus*. These spiders are commonly found on the ground wandering through the leaf litter, above ground in the vegetation of cottonwood trees (*Populus fremontii*) and desert willows (*Chilopsis linearis*), and occasionally in grass and on the dirt, rock, and gravel substrates (pers. obs).

### Data collection

Behavioral observations were conducted between 0900 and 1500 hrs. from March-November in 2009 and 2010 as part of a concurrent study on intraspecific courtship interactions (see [46]). We located spiders in the field by visually scanning the leaf litter and vegetation. When a spider was located, we conducted a two-part behavioral observation in which we followed the spider and observed their behavior directly, recording our observations using voice recorders. Sample sizes vary among species due to differences in abundance (*H*. *clypeatus*: n = 12 (5 adult females, 7 adult males), *H*. *hallani*: n = 14 (5 adult females, 4 adult males, 5 juveniles), *H*. *pyrrithrix*: n = 34 (15 adult females, 12 adult males, 7 juveniles), *H*. *hirsutus*: n = 27 (7 adult females, 15 adult males, 5 juveniles)). During the first 15 minutes of each observation, we quantified the amount of time the spider spent moving (i.e., walking, jumping) and stationary (i.e., not moving). While stationary, we quantified the amount of time spent in the sun versus the shade. In both the sun and shade, we further quantified the amount of time spent on different substrate types (cottonwood leaf litter, desert willow leaf litter, cottonwood vegetation, desert willow vegetation, grass, or rock/dirt). Finally, by marking the starting and ending location of the focal spider, we measured the total distance moved during the 15-minute observation period. While this ‘distance’ metric does not account for additional movement that did not occur in a straight line, those data are captured in the measurement of the amount of time the spider spent moving (see above).

If, after the 15-minute behavioral observation ended, the focal individual was *not* presently interacting with or oriented towards another individual, we conducted an additional 5-minute observation in which we quantified its leg-waving behavior. Outside of the context of courtship, individuals often raise and lower their front legs either simultaneously or in an alternating fashion (S1 File (video)). For each focal spider, we randomly selected either the left or right leg (to avoid the difficulty of observing both moving legs at the same time) and counted the number of times that leg was raised during the five-minute period. The reason that we counted leg-waves in a separate 5-minute observation period was that it was difficult to monitor leg-waves at the same time as monitoring the other behaviors described above.

After all data were collected, we temporarily captured each individual in a clear plastic vial. For putative adult females, we confirmed maturity by examining the epigynum; mature females can be distinguished from immatures by the presence of a sclerotized epigynum [55]. To ensure that we were not repeatedly observing the same individuals, we captured each spider after data were recorded and marked them with a small black dot (~1mm in diameter) on the underside of their abdomen using non-toxic black liquid eyeliner (Urban Decay Cosmetics, Costa Mesa, CA, USA) that produces a permanent mark. If, after collecting behavioral data for a given individual, we discovered that the individual had already been marked, we excluded those data for that individual from our analyses.

To better understand the suite of potential predators that may be shaping male and female behavior and/or color patterns, we recorded all predation events on *Habronattus* that we observed in the field throughout the study (n = 13), both within and outside of the context of our focal observations. Because we frequently saw several mud dauber wasps (family Sphecidae) in the area that we suspected were feeding on *Habronattus*, we also examined the contents of 23 abandoned mud dauber nests found on the underside of a cement bridge within the area where we carried out our spider behavioral observations. All of the wasp nests that we examined with emergence holes were empty, and thus here we include data only on the nests that we found that were sealed and did not have emergence holes. Wasp nests were likely inhabited by *Sceliphron* or *Chalybion* spp., both of which we have observed at this field site (pers. obs). Both species are specialist arachnivores; *Sceliphron* build mud nests in which they provision their young, while *Chalybion* use the old nests of *Sceliphron* (see [56,57–59]).

### Statistical analyses

All predictions above involve comparing behaviors between sexually mature males and females of the three species that exhibit conspicuous male coloration (*H*. *clypeatus*, *H*. *hallani*, and *H*. *pyrrithrix)*. For comparison with a species that does not exhibit conspicuous coloration, we also examined sex differences in behavior of the most distantly-related species, *H*. *hirsutus*. We used 2-tailed t-tests (if data met the relevant assumptions) or Wilcoxon rank sum tests (if assumptions of parametric tests were violated) for all comparisons. Note that we also collected data for a small number of immature spiders (see Data Collection section above); because our hypotheses don’t make explicit predictions about juvenile color patterns (the nature of which varies across the four species in both pattern and degree of sexual dimorphism) and because our sample sizes for juveniles was small and we were unable find juveniles of all four species, we excluded the juvenile data from our analyses. However we have archived it with the rest of our data in Dryad (doi:10.5061/dryad.v4h6741).

To test the initial predictions of the ‘deception hypothesis’, we compared leg-waving rates (number of leg-waves in five minutes) that occurred outside of the context of courtship between males and females. We then examined the hypotheses for why the sexes differ in their coloration strategies in *H*. *clypeatus*, *H*. *hallani*, and *H*. *pyrrithrix*. To test the predictions of the ‘activity hypothesis’, we compared the time spent actively moving (vs. time spent at rest) during behavioral observations between the sexes. To test the predictions of the ‘different microhabitat hypothesis’, we compared the time spent in each broad habitat category (leaf litter, vegetation, rock/dirt) and each finer-scale microhabitat category (cottonwood leaf litter, willow leaf litter, cottonwood vegetation, willow vegetation, grass, and rock/dirt) between the sexes. We then compared the time spent in different light environments (sun vs. shade) between the sexes. To understand if males and females differ in habitat heterogeneity, we compared the total distance traveled as well as the number of different microhabitat types through which individuals of each sex traveled during the observation period. Finally, we calculated the proportion of time that each individual spent in their preferred habitat type (i.e., the habitat type in which they spent the most time) and compared this between the sexes.

In this study, we were interested in broad-scale sex differences that could be replicated across sympatric species with conspicuous male color patterns (e.g., *H*. *clypeatus*, *H*. *hallani*, *H*. *pyrrithrix)*. In testing our hypotheses, we place emphasis on patterns that held up across species rather than emphasizing the importance of any one significant result for any one species; for this reason, we did not employ Bonferroni corrections (see [60]).

## Results

### Deception hypothesis

**Fig 3:**
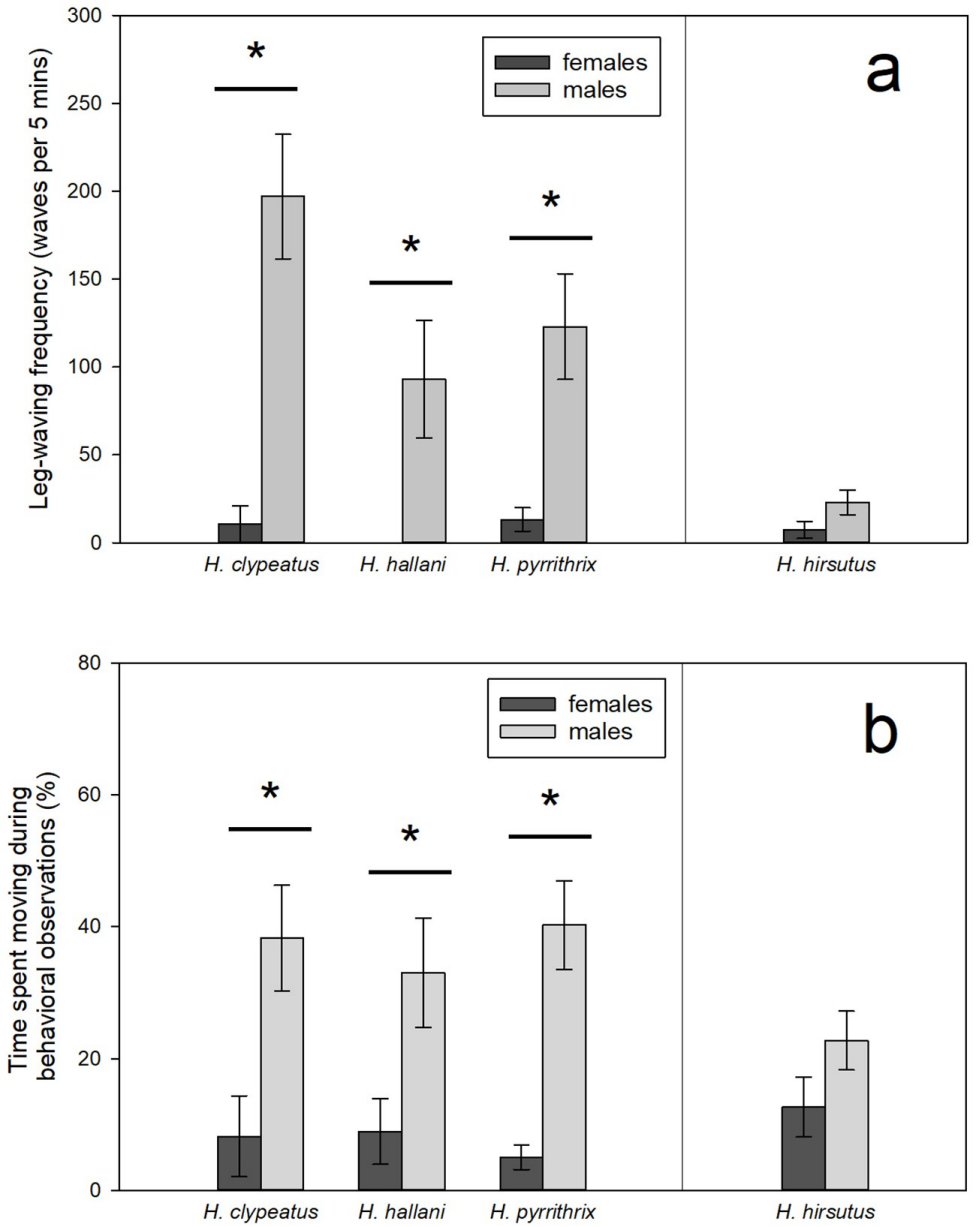
Comparisons of (a) non-courtship leg-waving frequency and (b) time spent moving between the sexes during behavioral observations in the field. Note that in the three conspicuously-patterned species (*H*. *clypeatus*, *H*. *hallani*, *and H*. *pyrrithrix*) males waved their legs more and spent more time moving than females. This pattern did not hold for *H*. *hirsutus*. Bars represent means (± SEM). Asterisks (*) indicate significant sex differences within a species.

In the three species with conspicuous male dorsal patterns, males waved their legs significantly more often than females (*H*. *clypeatus*: *t* = 5.04, *P* = 0.027; *H*. *hallani*: *X*^2^ = 4.76, *P* = 0.029; *H*. *pyrrithrix*: *X*^2^ = 9.47, *P* = 0.0021; Fig 3a). In *H*. *hirsutus*, males and females did not differ in leg-waving rates (*X*^2^ = 1.34, *P* = 0.25; Fig 3a).

### Activity hypothesis

In *H*. *clypeatus*, *H*. *hallani*, and *H*. *pyrrithrix*, males spent significantly more time moving than females (*H*. clypeatus: *t* = 2.98, *P* = 0.014; *H*. *hallani*: *t* = 2.49, *P* = 0.027; *H*. *pyrrithrix*: *X*^2^ = 17.20, *P*<0.0001; Fig 3b). For comparison, in *H*. *hirsutus*, males and females did not differ in movement rates (*X*^2^ = 2.52, *P* = 0.11; Fig 3b).

### Different microhabitat hypothesis

**Fig 4:**
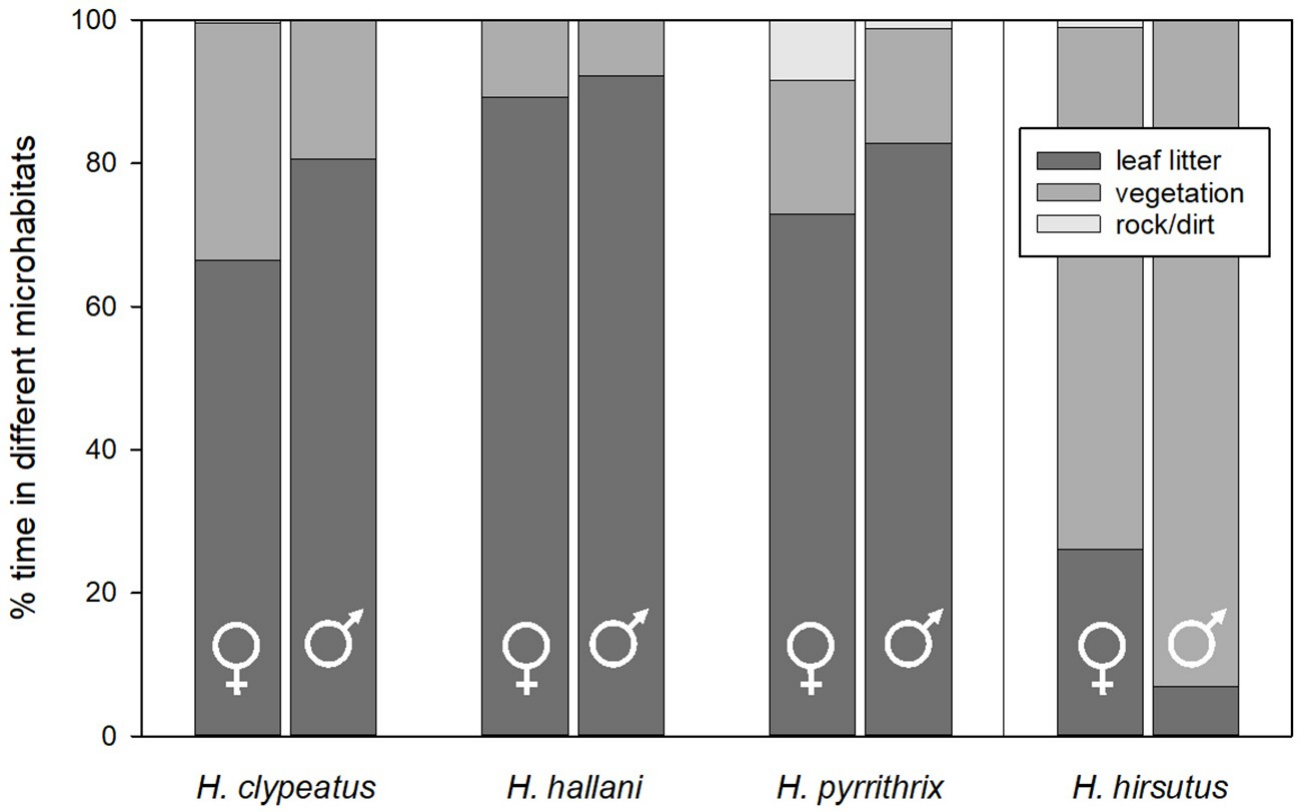
Comparison of mean proportions of time spent in different microhabitat types. Note that there are no significant sex differences with only one exception: in *H*. *hirsutus*, females spent more time resting on rock/dirt than males. For clarity, we include three broad habitat types in this analysis (leaf litter, vegetation, rock/dirt). However, in a second analysis, we broke up the microhabitats further by plant species (cottonwood leaf litter, desert willow leaf litter, cottonwood vegetation, desert willow vegetation, grass, and rock/dirt) and found the same patterns (S2 File).

In *H*. *clypeatus*, *H*. *hallani*, and *H*. *pyrrithrix*, males and females did not differ significantly in the amount of time they spent resting within the three major habitat types (*H*. *clypeatus*: leaf litter: *X*^2^ = 0.34, *P* = 0.56; vegetation: *X*^2^ = 0.34, *P* = 0.56; rock/dirt: *X*^2^ = 3.05, *P* = 0.081; *H*. *hallani*: leaf litter: *X*^2^ = 0.34, *P* = 0.56; vegetation: *X*^2^ = 0.34, *P* = 0.56; *H*. *pyrrithrix*: leaf litter: *X*^2^ = 0.38, *P* = 0.54; vegetation: *X*^2^ = 0.0024, *P* = 0.96; rock/dirt: *X*^2^<0.001, *P*>0.99; Fig 4). When the different habitat types were divided into finer categories (cottonwood litter, willow litter, cottonwood vegetation, willow vegetation, grass, and dirt/rock), there were still no sex differences for any habitat type in *H*. *clypeatus*, *H*. *hallani*, or *H*. *pyrrithrix* (S2 File). Finally, the sexes did not differ significantly in the amount of time spent in the sun vs. the shade (*H*. *clypeatus*: *X*^2^ = 1.00, *P* = 0.317; *H*. *hallani*: *X*^2^ = 2.23, *P* = 0.14; *H*. *pyrrithrix*: *X*^2^ = 0.40, *P* = 0.53).

For comparison, in *H*. *hirsutus*, there were no significant sex differences in the time spent resting on most substrate classes; the one exception was that females spent more time on rocks and dirt than males (leaf litter: *X*^2^ = 2.62, *P* = 0.11; vegetation: *X*^2^ = 2.62, *P* = 0.11; rock/dirt: *X*^2^ = 4.49, *P* = 0.034). When the microhabitat types were divided into finer categories as above, these patterns remained largely the same (S2 File). There was no difference between the sexes in how much time was spent in the sun vs. the shade (*X*^2^ = 2.30, *P* = 0.13).

In *H*. *pyrrithrix*, males moved greater distances than females (*H*. *pyrrithrix*: *X*^2^ = 4.99, *P* = 0.026), and there was a nonsignificant trend in the same direction for *H*. *hallani* (*H*. *hallani*: *X*^2^ = 3.53, *P* = 0.060). In contrast, in *H*. *clypeatus*, there was no sex difference in the total distance traveled over the course of behavioral observations (*H*. *clypeatus*: *t* = 0.90, *P* = 0.40). Despite data suggesting that males of some species travel further than females, there were no sex differences in the number of microhabitat types moved through during behavioral observations for any of the species (*H*. *clypeatus*: *t* = -0.97, *P* = 0.36; *H*. *hallani*: *X*^2^ = 0.80, *P* = 0.37; *H*. *pyrrithrix*: *X*^2^ = 0.28, *P* = 0.60). Furthermore, males and females did not differ in the amount of time they spent in their preferred habitat type (*H*. *clypeatus*: *t* = -0.65, *P* = 0.53; *H*. *hallani*: *X*^2^ = 0.34, *P* = 0.56; *H*. *pyrrithrix*: *X*^2^ = 0.022, *P* = 0.88).

In *H*. *hirsutus*, males and females did not differ in total movement distance (*X*^2^ = 1.11, *P* = 0.29), number of microhabitats used (*X*^2^ = 3.11, *P* = 0.078), or in the amount of time spent in their preferred habitat type compared with other habitats (*X*^2^ = 1.94, *P* = 0.16).

### Predation on *Habronattus*

We directly observed thirteen predation events on *Habronattus* over the course of the study (Table 1). Seventy-seven percent of these involved predation by conspecifics (n = 6) or heterospecific *Habronattus* (n = 4); the other three were by an ant, a wolf spider, and a different salticid species (Table 1). Of the *Habronattus* events, the predators were always the same size or larger than the prey; in 80% of cases, the predators were adult females, while the remaining 20% were juveniles (Table 1).

**Table 1:** List of predation events on *Habronattus* observed at our field site.

Prey species	Prey sex	Size class (mm)	Predator	Predator sex	Size class (mm)
*H*. *clypeatus*	adult male	5	*H*. *clypeatus*	adult female	6
	adult male	5	*H*. *pyrrithrix*	adult female	6
	adult male	5	*H*. *pyrrithrix*	adult female	6
*H*. *hirsutus*	adult female	6	*H*. *hallani*	adult female	6
	juvenile*	3	*H*. *hallani*	adult female	6
	juvenile*	3	*H*. *hirsutus*	juvenile*	3
	juvenile*	4	*H*. *hirsutus*	adult female	6
	juvenile*	4	*H*. *hirsutus*	juvenile*	4
	juvenile*	4	*H*. *hirsutus*	adult female	6
*H*. *pyrrithrix*	adult female	2	Formicidae (ant)	unknown	3
	adult female	6	Lycosidae (wolf spider)	adult female	10
	adult male	5	*H*. *pyrrithrix*	adult female	6
	adult male	5	*Phidippus* sp. (Salticidae)	juvenile*	7

* In *H*. *hirsutus* and *Phidippus* sp., sex-specific color patterns do not appear until maturity; thus, the sex of young juveniles (before the penultimate stage of development) is unknown

**Fig 5:**
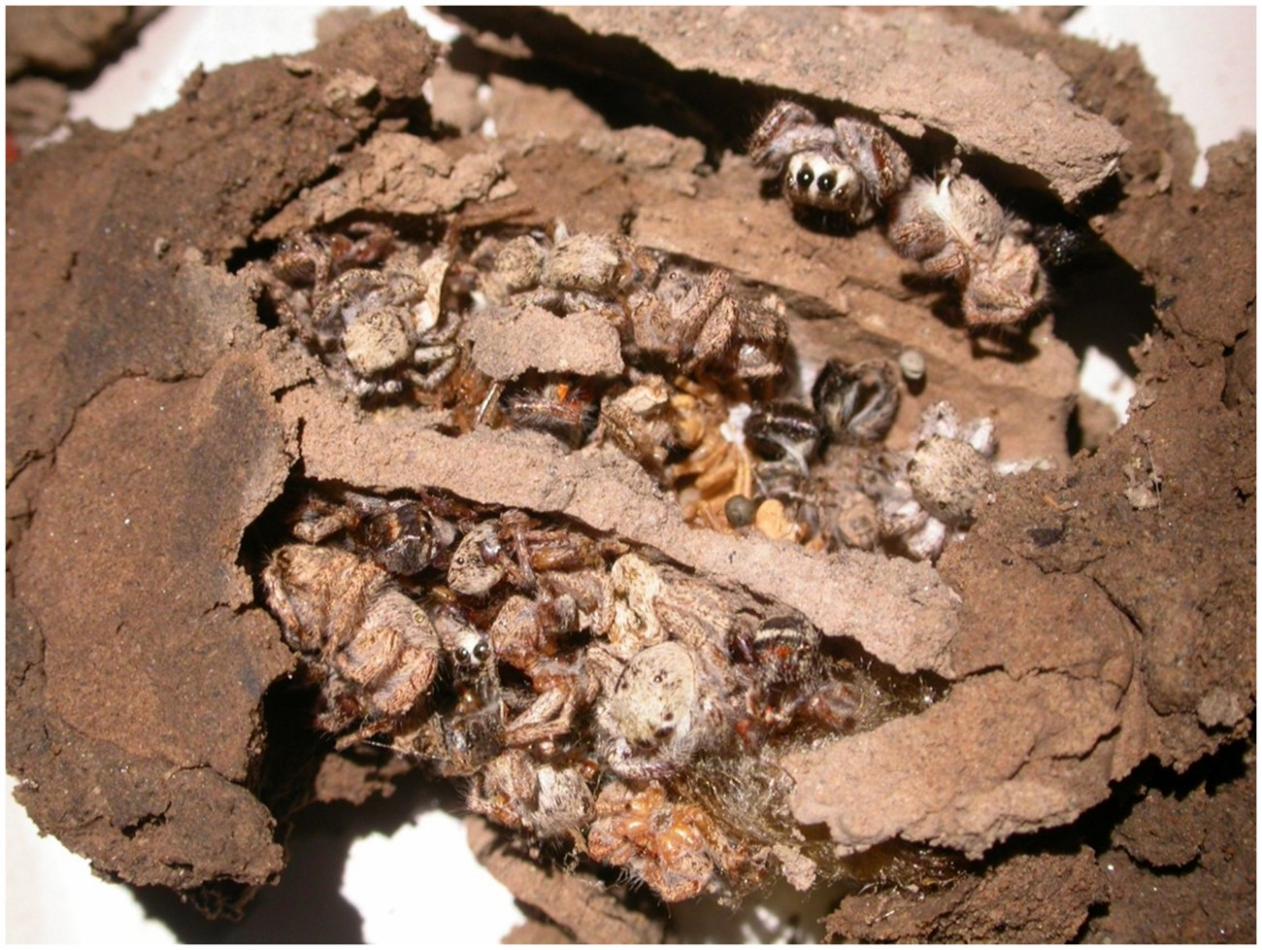
Contents of three cells of a mud-dauber wasp nest (Sphecidae) found at our field site. The nest contents indicate that, at least for some individual wasps, *Habronattus* makes up a significant portion of their prey.

By examining the remains of old mud dauber wasp nests (n = 23), we found that salticids, and in particular *Habronattus*, make up a significant part of the wasp diet (Table 2, Fig 5). Of the 23 inspected nests, 14 still had remaining spider provisions; the other 9 had dead wasps (adults, larvae, or pupae) that appeared to have eaten all of their provisions but for some reason did not survive to emergence. Of the nests that still had spider provisions remaining, the total number of spiders per cell ranged from 4 to 51, with a total of 211 spiders, 31 of which were salticids. Three of the fourteen nests contained at least one salticid. Interestingly, for the nests that contained any salticids at all, 75% of the spiders that were in those nests were salticids. Of those salticids, 89% (n = 26) were *H*. *hirsutus* while the rest were immature *Phidippus* sp. All *H*. *hirsutus* specimens found in nests were mature adults (n = 12) or large juveniles (≥ 4mm, n = 14) (Table 2).

**Table 2:** *Habronattus* specimens found in mud dauber wasp nests (family Sphecidae) at our field site.

Prey species	Sex	Size class (mm)	Quantity
*H*. *hirsutus*	adult female	6	10
*H*. *hirsutus*	adult male	6	2
*H*. *hirsutus*	subadult male†	5	2
*H*. *hirsutus*	juvenile*	4	12

^†^ For all *Habronattus*, subadult males (just prior to sexual maturity), exhibit enlarged pedipalps, allowing us to identify them as males before their final molt into adult coloration

* In *H. hirsutus*, sex-specific color patterns do not appear until maturity; thus, the sex of most juveniles is unknown

## Discussion

From direct observations of free-ranging spiders in the field, we show that conspicuous sexually dimorphic dorsal patterns in males of three species of *Habronattus* (*H*. *clypeatus*, *H*. *hallani*, and *H*. *pyrrithrix*) were associated with increased male leg-waving behavior, which is qualitatively similar to the false antennation behavior exhibited by other spiders that mimic hymenopterans (e.g., [6,7]). Females of all three species are cryptic in coloration and do not engage in this putative false antennation behavior. Also consistent with the deception hypothesis is the finding that, in the one species in our study in which males do not have conspicuous markings (*H*. *hirsutus*), males correspondingly did not show the increased leg-waving behaviors seen in the other three species. When examining two hypotheses for why males and females from three species differ in their dorsal coloration strategies, we found support for the activity hypothesis, which posits that higher movement rates of males may have constrained the evolution of cryptic coloration, resulting in different dorsal colors in the two sexes. Because sexes did not differ in microhabitat use, our data failed to support the different habitat hypothesis for sex differences in coloration (discussed in more detail below).

### Evidence for deceptive coloration and its alternatives in *Habronattus* males

While our observations and field data are consistent with and provide preliminary support for the idea that dorsal color and behavior work together in *Habronattus* to deceive predators, it is important to weigh support against alternative explanations that are commonly proposed for conspicuous colors, such as disruptive coloration (e.g., [32,33,34]) or motion dazzle markings [35–37,61]. Disruptive coloration is a form of camouflage that employs markings that break up an animal’s outline or create the appearance of false edges and boundaries, hindering the detection or recognition of an animal’s true shape (e.g., [32,33,34]). As such, disruptive pattern elements are expected to be biased towards borders and edges [62]; empirical work has shown that such markings are indeed more effective at deterring predation when they occur along body outlines [63,64]. In the conspicuously colored *Habronattus* males in our study, the contrasting patterns are located in the center of the abdomen (Fig 1). In contrast with disruptive coloration, which interrupts edges, in all three species, the abdomen is bordered with a conspicuous black or white margin, which clearly highlights the outline of the body, rather than disrupting it (Fig 1a, 1c and 1e). Furthermore, the frequent leg-waving of males attracts attention and clearly identifies the head of the spider, which appears to emphasize and clarify the body shape rather than obscuring it (pers. obs), suggesting that this patterning is not functioning as disruptive coloration.

Another potential explanation for conspicuous markings that has received recent attention is the idea that they interact with movement creating a ‘motion dazzle’ effect, making it difficult for predators to estimate the speed and trajectory of their prey at high speeds [35,36,61]. Empirical support comes from human subjects and computer animations; at high speeds, dazzle coloration affects the perceived speed of objects moving in a straight line [36] and makes the capture of moving prey more difficult [35,37]. This, too, seems improbable for *Habronattus* males, whose movement patterns are relatively slow compared with the animations used in dazzle studies (15-20cm/s [35], 357 cm/s [36]). Furthermore, movement patterns of *Habronattus* males are jerky, and involve frequent stopping and zig-zagging through their habitat as they search for females (pers. obs), making this an unlikely system for dazzle coloration to be effective.

The present study provides a first step towards understanding if and how male dorsal patterns in *Habronattus* may deceive predators. An important next step will be to design experiments using color-manipulated spiders (or models or videos where leg-waving behavior could be manipulated) and ecologically relevant suites of predators to further examine the ideas we present here. Our study examines color patterns in their natural context with an emphasis on natural history; it has been argued that this emphasis is missing from many studies on protective coloration [61]. Furthermore, our results identify two groups of organisms that are likely to be important predators in this system (mud dauber wasps that specialize on spiders and other *Habronattus*), setting the stage for work that examines how predator perception and cognition influence the evolution of prey color patterns and behaviors (e.g., [61,65]). Because mud dauber wasps hunt exclusively for spiders (e.g., [56,57–59,66]) and never the hymenopterans that *Habronattus* males appear to mimic, the deceptive strategy of male *Habronattus* may simply allow them to escape a mud dauber’s search image, rather than to perfectly mimic any one type of wasp or bee. While we know little about the particular color and pattern preferences of any mud dauber wasp, we do know that *Sceliphron* mud daubers individually specialize on spider prey, with a single individual wasp sometimes taking up to 25 individuals of the same species of spider in the same day [66]; as such, simply escaping a mud dauber’s search image for spiders may be critically important.

Interestingly, in the single species in our study in which males do not exhibit conspicuous coloration (*H*. *hirsutus*), males correspondingly lacked the increased leg-waving behavior relative to females. While this is consistent with the idea that male color pattern and leg-waving function together in *Habronattus*, a comparative study that examines a larger number of species both with and without conspicuous coloration is clearly needed. The genus *Habronattus* is both diverse and speciose [27], and the availability of a molecular phylogeny [38] makes it an ideal group for testing hypotheses about the coevolution of dorsal color pattern and leg-waving behavior within a phylogenetic framework. Such comparative studies may also help explain why males of different species, such as *H*. *hirsutus*, have not adopted conspicuous color patterns like those found in *H*. *clypeatus*, *H*. *hallani*, and *H*. *pyrrithrix* males. Data from this study indicate that *H*. *hirsutus* spends 87% of their time above ground in the vegetation compared with the other three species, which all spend more than 74% of their time in the leaf litter on the ground (Fig 4). Furthermore, *H*. *hirsutus* was the only species of *Habronattus* that we found in mud dauber nests (Table 2); anecdotally, we more often saw mud-daubers foraging in the trees than we did on the ground at our field site (pers. obs.). Such differences in natural history should be explored further as potential explanations for the selection pressures that have differently shaped male color patterns in these spiders.

We tested two hypotheses to explain why male and female *H*. *clypeatus*, *H*. *hallani*, and *H*. *pyrrithrix* differ in dorsal color pattern. We failed to uncover support for the different habitat hypothesis. In none of these species did males and females differ in the time spent in different microhabitats, with only one exception: *H*. *hirsutus* females spent more time on dirt and rock compared with males. None of the species differed in the amount of time that the sexes spent in the sun vs. the shade. While there was some evidence that males traveled greater distances than females during observations, this did not lead to males traversing a larger number of habitat types during observations. Furthermore, there were no differences in the proportion of time spent in an individual’s preferred habitat type relative to other habitat types. Instead, the activity hypothesis to explain male color pattern differences was supported in all three conspicuously colored species; males indeed spent more time actively moving than females. Interestingly, and as expected, this relationship did not hold up for *H*. *hirsutus*.

### A novel twist on the activity hypothesis

Here we showed that, in three species of *Habronattus*, females spent relatively small amounts of time moving compared to males, and thus, cryptic coloration may be an ideal strategy. In contrast, males move more, presumably in search of females, and thus they must find an alternative strategy for protection. Our study lends preliminary support to the idea that males may have solved this problem by pairing conspicuous patterns of bold stripes and chevrons with leg-waving behavior to subtly resemble wasps or bees and thus deceive predators. The rationale behind the activity hypothesis is not new; both theoretical and empirical work support the idea that movement can limit the effectiveness and ultimately constrain the evolution of cryptic coloration, leading to alternative solutions for protection in other animals (e.g., [39,42,43]).

Our evidence in support of the activity hypothesis comes from observations on males outside of the context of courtship, as leg-waving behavior was only quantified when males were not interacting with other spiders (see Methods). Yet observations of male courtship behavior suggest that the same color patterns that presumably protect males when wandering in search of females might also be strategically designed to function while males are actively courting. Males must use extreme caution when displaying for females who are also predators and often attack conspecifics (e.g., see Table 1, see also [46,67]). Given this risk, it is not surprising that a courting male *Habronattus* is extremely focused on the female during courtship and often appears oblivious to external stimuli or threats when courting (pers. obs., see also [45]). Here we propose the ‘focused courtship’ hypothesis, as an extension of the activity hypothesis, to account for a potentially added benefit of combining leg-waving during courtship with mimetic dorsal coloration. Because males must already extend and wave their first pair of legs to communicate with females during courtship, adding conspicuous patterning to their backs creates the combination of conspicuous stripes and leg-waving that is characteristic of hymenopteran mimics. It has been suggested that, in ant mimics, behavioral mimicry (i.e., false antennation) evolved before morphological mimicry (reviewed in [7]). In *Habronattus*, as well as other salticids, this transition to behavioral mimicry might be a relatively simple step, as it involves taking a behavior that is already part of courtship (leg-waving) and extending it to the mate-search context as well. In courting male *Habronattus* jumping spiders, actively and clearly identifying their location to females from a safe distance is a necessity; this goal likely conflicts with any attempt to avoid detection by potential predators (which include the very females whose attention they are trying to capture). As such, selection on *Habronattus* should drive protective strategies that continue to work *after* detection and that do not require the male to risk taking his focus off of a potentially cannibalistic female; this may explain why mimicry is so widespread across Salticidae (e.g., [7]).

In conclusion, here we provide preliminary evidence consistent with the idea that, in three sympatric species of *Habronattus*, male color and pattern may work together to deceive predators, an idea that should be further examined with manipulative experiments. We also suggest that dorsal coloration differs between males and females due to sex differences in activity patterns (i.e., movement rates) and speculate that imperfect mimetic dorsal coloration provides an added anti-predator benefit to males while they intently court a female. Despite the extensive literature on the function and evolution of protective coloration [15,30,33,61,68], to our knowledge no study has examined sex differences in non-display coloration in Salticidae. This is surprising, given the degree to which non-display dorsal color patterns differ between the sexes across the family, often being more conspicuous in males (e.g., see [17]). While male leg-waving and striped patterns certainly do not cause male *Habronattus* to perfectly resemble a particular wasp or bee species, these traits may be a form of imperfect mimicry exploiting a potential predator’s innate aversions [69]. In the case of avoiding attack from spider-hunting mud dauber wasps, simply looking like anything other than a spider might be enough to deter an attack. Such systems may provide new insights into the function and evolution of diversity in animal color patterns.

## Supporting information

S1 Fig Examples of potential wasp mimicry in jumping spiders ranging from near perfect to imperfect mimicry. In *Phiale formosa*, males (a) and females (b) both appear to be mimics, but clearly use different strategies; males have a striking black and white color pattern similar to velvet ants in the area, while females are black and yellow and appear to be general wasp mimics. These females don’t appear to mimic any one particular wasp species, but instead appear to rely on a general resemblance to wasps. The jumping spider *Phiale mimica* (c) bears a striking resemblance to the velvet ant *Dasymutilla cressonii* (d) that is found in the same area.
(TIF)

S1 File (Video). Video showing male leg-waving behavior in *Habronattus pyrrithrix*. Even in the absence of another spider, wandering males wave their legs while moving through their habitat. Here we propose that this leg-waving behavior (more pronounced in males than females, see Results) works with their conspicuous dorsal patterns to deceive predators.
(MP4)

S2 File (Table) Results of Wilcoxon rank sum tests examining sex-differences in microhabitat use in four species of *Habronattus* jumping spiders. Dashes (-) denote microhabitat types in which a given species was never found.
(PDF)
