# Supplementary figures and images for: Variation in activity rates may explain sex-specific dorsal color patterns in Habronattus jumping spiders
Source: PLoS One. 2019 Oct 16;14(10):e0223015. doi: 10.1371/journal.pone.0223015 (PMC6795386; doi:10.1371/journal.pone.0223015)

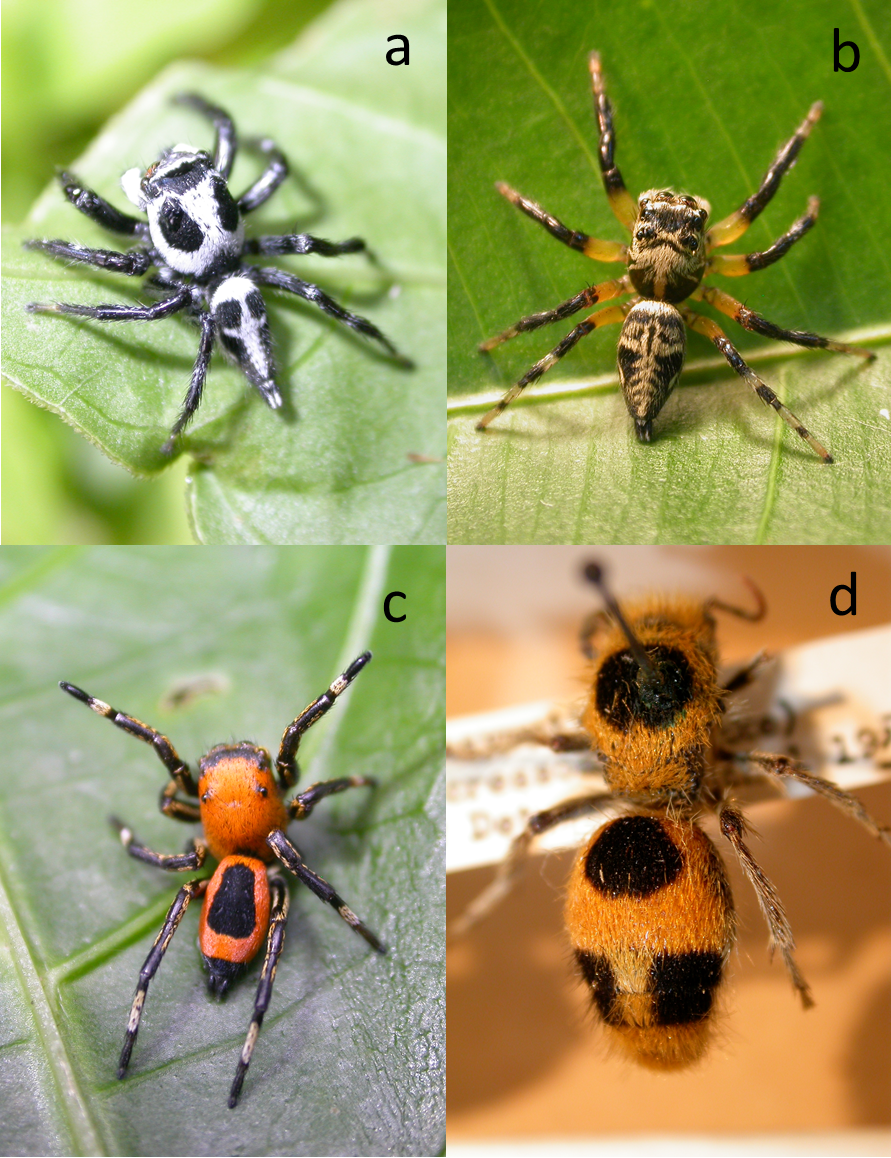

Supplement: S1 Fig — In Phiale formosa, males (a) and females (b) both appear to be mimics, but clearly use different strategies; males have a striking black and white color pattern similar to velvet ants in the area, while females are black and yellow and appear to be general wasp mimics. These females don’t appear to mimic any one particular wasp species, but instead appear to rely on a general resemblance to wasps. The jumping spider Phiale mimica (c) bears a striking resemblance to the velvet ant Dasymutilla cressonii (d) that is found in the same area. (TIF) [file pone.0223015.s001.tif]
