# Supplementary material for: Variation in activity rates may explain sex-specific dorsal color patterns in Habronattus jumping spiders
Source: PLoS One. 2019 Oct 16;14(10):e0223015. doi: 10.1371/journal.pone.0223015 (PMC6795386; doi:10.1371/journal.pone.0223015)
Supplement: S2 File — Dashes (-) denote microhabitat types in which a given species was never found. (PDF) [file pone.0223015.s003.pdf]

S2 File (Supplemental Table)

Taylor LA, Cook CD, and McGraw KJ. Sex differences in protective coloration: variation in activity rates may explain dorsal color patterns in *Habronattus* jumping spiders

Results of Wilcoxon rank sum tests examining sex-differences in microhabitat use in four species of *Habronattus* jumping spiders. Dashes (-) denote microhabitat types in which a given species was never found.

Species	df	X^2	*P*
H. clypeatus			
cottonwood leaf litter	1	0.18	0.67
willow leaf litter	1	0.04	0.85
cottonwood vegetation	1	1.56	0.21
willow vegetation	1	0.34	0.56
grass	1	1.40	0.24
dirt/rock	1	3.05	0.08
H. hallani			
cottonwood leaf litter	1	0.34	0.56
willow leaf litter	1	-	-
cottonwood vegetation	1	0.34	0.56
willow vegetation	1	-	-
grass	1	-	-
dirt/rock	1	-	-
H. pyrrithrix			
cottonwood leaf litter	1	0.82	0.36
willow leaf litter	1	2.02	0.16
cottonwood vegetation	1	0.016	0.90
willow vegetation	1	0.012	0.91
grass	1	1.25	0.26
dirt/rock	1	<0.001	>0.99
H. hirsutus			
cottonwood leaf litter	1	2.62	0.11
willow leaf litter	1	<0.001	>0.99
cottonwood vegetation	1	0.47	0.49
willow vegetation	1	3.62	0.057
grass	1	2.14	0.14
dirt/rock	1	4.49	**0.034**

Significant *P* values are shown in bold.
